# Serum Vitamin D Levels in Patients With Distal Radius Fractures Presenting to a Tertiary Care Hospital

**DOI:** 10.7759/cureus.21749

**Published:** 2022-01-30

**Authors:** Muhammad Amin Chinoy, Kazim Hussain, Salman Javed

**Affiliations:** 1 Orthopaedics, The Indus Hospital Karachi, Karachi, PAK

**Keywords:** alkaline phosphate, calcium, fractures, distal radius, vitamin d

## Abstract

Objective

To compare the frequency of serum vitamin D levels in patients with and without distal radius fracture.

Introduction

Hypovitaminosis D is a common problem worldwide. Deficiency of vitamin D is not only associated with musculoskeletal problems, but also with chronic diseases like diabetes mellitus, cardiovascular disease and cancer. Vitamin D deficiency results in bone pain, aggravating osteoporosis and fragility fractures. Distal radius fractures are common upper limb fractures, mostly in the elderly population. Low serum levels of vitamin D have been reported to result in increased risk of distal radius fracture.

Methodology

This study was conducted at The Indus Hospital Karachi, from 1st March 2020 to 31st August 2020. Consecutive non-probability sampling was done. Patients aged between 20 to 80 years with and without distal radius fractures were enrolled and serum vitamin D levels were compared.

Results

A total of 130 patients were enrolled in this study, out of which 70 (53.8%) were males. The median (IQR) vitamin D, calcium, alkaline phosphate level, and albumin was 14.8 (9.5-23) ng/ml, 9.2 (8.9-9.5) ng/ml, 91 (77.3-111) U/L, and 4.2 (4-4.5) g/dl, respectively. No significant association between distal radius fracture and vitamin D levels was found in males, whereas a significantly higher proportion of females with no fracture had a mild-moderate deficiency in comparison to females with fracture.

Conclusion

No significant association was noted between patients with and without distal radius fracture and vitamin D levels, however, we recommend more studies on this topic so that more comprehensive results can be obtained.

## Introduction

Vitamin D (Vit D) deficiency and insufficiency are common problems worldwide and in Pakistan [1]. Serum levels of less than 20ng/ml are termed a deficiency, while levels between 20ng/ml and 29.9ng/ml are insufficient [2]. Low levels of Vit D cause musculoskeletal problems and are also associated with chronic diseases like diabetes mellitus, cardiovascular disease, and cancer [3]. Age, gender, low exposure to sunlight, inadequate dietary Vit D, and lower socioeconomic status are associated with Vit D deficiency and insufficiencies [1]. Vit D deficiency is directly associated with bone pain, aggravating osteoporosis, and fragility fractures [4]. Vit D regulates over 200 genes in the human body [5]. Serum 25-hydroxyvitamin D level is the best marker of Vit D status [6, 7].

A major source of Vit D is sunlight, which converts 7-dehydrocholesterol in the stratum spinosum and stratum basale of the skin's epidermis to pre-cholecalciferol, which is further converted to form cholecalciferol. Cholecalciferol undergoes the first hydroxylation in the liver and then the second hydroxylation in kidneys to form the active form of vitamin D [8]. Socio-cultural factors such as confinement to indoor, pardah culture, and economic factors resulting in insufficient micronutrients from dietary sources are postured to be the reason for low Vit D in our country [8].

Vit D deficiencies in patients with osteoporotic fractures are common [9], especially in elderly individuals. Literature has shown that daily supplements of Vit D and adequate levels reduce the risk of falling and fragility fractures [6] and reduce the risk of a second fracture within three years [10] mainly in the elderly population, however, data regarding the younger age group is scantly available.

Distal radius fractures are the common upper limb fractures [11], and in recent years the incidence of the distal radius has increased worldwide [12]. With low serum levels of Vit D in the elderly population, there is an increased risk of distal radius fracture [6] and a subsequent hip or spine fracture [10]. However, irrespective of age, the association between Vit D deficiency and distal radius fracture is not reported. Therefore, we set out to compare Vit D levels in patients presenting with distal radius fracture and a control group to determine the role of Vit D in distal radius fractures. To the best of our knowledge, no study has been conducted to evaluate serum levels of Vit D in patients with distal radius fractures in our region. This can help us identify patients at an increased risk of fractures in the future and can be referred to a specialist clinic for assessment, evaluation, and therapy to prevent many other complications.

## Materials and methods

The study was conducted at The Indus Hospital Karachi, a tertiary care hospital, from 1st March 2020 to 31st August 2020 after approval of the ethical review board. The sample size was calculated using the WHO sample size calculator and 130 patients using the assumptions, frequency of Vitamin D insufficiency reported previously 31.4% (Level of confidence 95% and margin of error 8%). Consecutive non-probability sampling was done on patients visiting emergency and outpatient departments.

The participants included were between age 20 and 80 years of both genders. Two groups were made, one group had a history of fall or a history of trauma with distal radius fracture (intraarticular and extra-articular fracture), diagnosed using X-rays, and second group patients who do not have a distal radius fracture presenting for some other orthopaedic-related issue to The Indus Hospital, Karachi. Patients with any known metabolic disorders (e.g., rickets, osteomalacia, hyperparathyroidism, chronic kidney disease, etc.), open fractures, pregnant women, and those already on vitamin D and calcium supplements during the last three months were excluded from the study. Once an eligible participant was identified, the study details were carefully discussed, and informed consent was taken. After ensuring the consent, a questionnaire was filled which includes serial number, age, medical record number, gender, any other supplements intake during the last three months, comorbidities (as verified from their history), serum Vit D levels, and levels of other secondary tests (Calcium, Alkaline Phosphatase, and Albumin). All the investigations were done routinely in the hospital without any charges from the patients.

Data was entered and analyzed using SPSS version 24.0 (IBM Corp., Armonk, NY). Mean ± SD/Median (IQR) was computed for all the quantitative variables like age, height, weight, BMI, serum Vit D, calcium, alkaline phosphatase, albumin, and duration of sun exposure per day. Frequency and percentage were computed for all the categorical variables like gender, occupation, comorbidities, smoking status, and menopause. Effect modifiers were controlled through stratification of age, gender, BMI, comorbidity, smoking status, duration of sun exposure per day, albumin, and menopause. P-value<0.05 was considered statistically significant.

## Results

A total of 130 patients were enrolled in this study, out of which 70 (53.8%) were males. The median (IQR) of age and BMI of all the participants were 41 (30.8-51) years and 25.5 (23.7-28) kg/m^2^, respectively. The majority of the participants were employed (n=51; 41.1%), and 49 (39.5%) were housewives (Table 1).

**Table 1:** Demographic characteristics of participants (n=130)

Age (Years)	
Min-Max	20-79
Median (IQR)	41 (30.8-51)
Weight (kg)	
Min-Max	44-120
Median (IQR)	75 (67-80)
Height (cm)	
Min-Max	117-183.5
Median (IQR)	170 (161.8-175)
BMI (kg/m^2^)	
Min-Max	16.1-46.6
Median (IQR)	25.5 (23.7-28)
Duration of sun exposure (hours)	
Min-Max	0.1-9
Median (IQR)	1 (0.5-2)
Gender; n (%)	
Male	70 (53.8)
Female	60 (46.2)
Total	130 (100)
Menopause (for females); n (%)	
No	44 (33.8)
Yes	16 (12.3)
Missing	70 (53.8)
Total	130 (100)
Addiction; n (%)	
Smoker	16 (12.3)
Occasional Smoker	12 (9.2)
Non-Smoker	102 (75.5)
Total	130 (100)
Comorbidity; n (%)	
No	81 (62.3)
Yes	49 (37.7)
-Diabetes	14 (10.4)
-Hypertension	23 (17)
-Other	17 (12.6)
Distal Radius Fracture; n (%)	
Extra-articular	31 (23.8)
Intra-articular	34 (26.2)
No distal radius fracture	65 (50)
Total	130 (100)
Occupation; n (%)	
Employed	51 (41.1)
Unemployed	12 (9.7)
Housewife	49 (39.5)
Students	12 (9.7)

It was observed that the participants had very minimal exposure to the sun [Median (IQR): 1 (0.5-2) hours] (Table 1). However, the median (IQR) Vit D, calcium, alkaline phosphate level, and albumin were 14.8 (9.5-23) ng/ml, 9.2 (8.9-9.5) ng/ml, 91 (77.3-111) U/L, and 4.2 (4-4.5) g/dl, respectively (Table 2).

**Table 2:** Laboratory findings of the participants

Vitamin D level (ng/ml)	
Min-Max	3.8-154.2
Median (IQR)	14.8 (9.5-23)
Calcium level (mg/dl)	
Min-Max	3.7-10.2
Median (IQR)	9.2 (8.9-9.5)
Alkaline Phosphatase level (U/L)	
Min-Max	42-470
Median (IQR)	91 (77.3-111)
Albumin level (g/dl)	
Min-Max	2.3-9.8
Median (IQR)	4.2 (4-4.5)
Vitamin D level	n%
Severe deficiency	33 (25.4)
Normal	72 (55.4)
Mild to moderate deficiency	22 (16.9)
Toxicity	3 (2.3)
Total	130 (100)

Furthermore, no significant association between distal radius fracture and Vit D levels was found in males. A significantly higher proportion of females with no fracture had a mild-moderate deficiency than females with fracture (n=9; 29% vs. 1; 3.4%, p=0.032, Table 3). A similar pattern was seen in patients of age ≤41 years, BMI ≤25 kg/m^2^, females with no menopause, and albumin >4.2. Also, the results were statistically significant (p=0.012, 0.003, 0.014 and 0.010, respectively, Table 3).

**Table 3:** Association of vitamin D levels with distal radius fracture post-stratification of various participants’ characteristics

Participants' characteristics	Vit D levels	Distal Radius Fracture		Total	p-value
		Yes	No		
		n (%)	n (%)		
Gender					
Male	Severe deficiency	6(16.7)	5(14.7)	11(15.7)	0.116ꬸ
	Normal	27(75)	19(55.9)	46(65.7)	
	Mild to moderate deficiency	3(8.3)	9(26.5)	12(17.1)	
	Toxicity	0(0)	1(2.9)	1(1.4)	
	Total	36(100)	34(100)	70(100)	
Female	Severe deficiency	11(37.9)	11(35.5)	22(36.7)	0.032*ꬸ
	Normal	16(55.2)	10(32.3)	26(43.3)	
	Mild to moderate deficiency	1(3.4)	9(29)b	10(16.7)	
	Toxicity	1(3.4)	1(3.2)	2(3.3)	
	Total	29(100)	31(100)	60(100)	
Age in years					
≤41	Severe deficiency	9(30)	9(24.3)	18(26.9)	0.012*ꬸ
	Normal	21(70)	18(48.6)	39(58.2)	
	Mild to moderate deficiency	0(0)	8(21.6)b	8(11.9)	
	Toxicity	0(0)	2(5.4)	2(3)	
	Total	30(100)	37(100)	67(100)	
>41	Severe deficiency	8(22.9)	7(25)	15(23.8)	0.067ꬸ
	Normal	22(62.9)	11(39.3)	33(52.4)	
	Mild to moderate deficiency	4(11.4)	10(35.7)	14(22.2)	
	Toxicity	1(2.9)	0(0)	1(1.6)	
	Total	35(100)	28(100)	63(100)	
BMI category					
≤25	Severe deficiency	9(29)	4(13.8)	13(21.7)	0.003*ꬸ
	Normal	21(67.7)	15(51.7)	36(60)	
	Mild to moderate deficiency	0(0)	9(31)b	9(15)	
	Toxicity	1(3.2)	1(3.4)	2(3.3)	
	Total	31(100)	29(100)	60(100)	
>25	Severe deficiency	8(23.5)	12(33.3)	20(28.6)	0.112ꬸ
	Normal	22(64.7)	14(38.9)	36(51.4)	
	Mild to moderate deficiency	4(11.8)	9(25)	13(18.6)	
	Toxicity	0(0)	1(2.8)	1(1.4)	
	Total	34(100)	36(100)	70(100)	
Menopause (for females)					
No	Severe deficiency	9(42.9)	8(34.8)	17(38.6)	0.014*ꬸ
	Normal	12(57.1)	7(30.4)	19(43.2)	
	Mild to moderate deficiency	0(0)	7(30.4)B	7(15.9)	
	Toxicity	0(0)	1(4.3)	1(2.3)	
	Total	21(100)	23(100)	44(100)	
Yes	Severe deficiency	2(25)	3(37.5)	5(31.3)	1.000ꬸ
	Normal	4(50)	3(37.5)	7(43.8)	
	Mild to moderate deficiency	1(12.5)	2(25)	3(18.8)	
	Toxicity	1(12.5)	0(0)	1(6.3)	
	Total	8(100)	8(100)	16(100)	
Sun exposure in hours					
≤1 hour	Severe deficiency	10(26.3)	12(29.3)	22(27.8)	0.316ꬸ
	Normal	23(60.5)	18(43.9)	41(51.9)	
	Mild to moderate deficiency	4(10.5)	10(24.4)	14(17.7)	
	Toxicity	1(2.6)	1(2.4)	2(2.5)	
	Total	38(100)	41(100)	79(100)	
> 1 hour	Severe deficiency	7(25.9)	4(16.7)	11(21.6)	0.003*ꬸ
	Normal	20(74.1)	11(45.8)b	31(60.8)	
	Mild to moderate deficiency	0(0)	8(33.3)b	8(15.7)	
	Toxicity	0(0)	1(4.2)	1(2)	
	Total	27(100)	24(100)	51(100)	
Albumin					
≤4.2	Severe deficiency	9(28.1)	12(30.8)	21(29.6)	0.253ꬸ
	Normal	19(59.4)	16(41)	35(49.3)	
	Mild to moderate deficiency	3(9.4)	10(25.6)	13(18.3)	
	Toxicity	1(3.1)	1(2.6)	2(2.8)	
	Total	32(100)	39(100)	71(100)	
>4.2	Severe deficiency	8(24.2)	4(15.4)	12(20.3)	0.010*ꬸ
	Normal	24(72.7)	13(50)	37(62.7)	
	Mild to moderate deficiency	1(3)	8(30.8)b	9(15.3)	
	Toxicity	0(0)	1(3.8)	1(1.7)	
	Total	33(100)	26(100)	59(100)	
Calcium level					
≤9.2	Severe deficiency	10(32.3)	11(28.9)	21(30.4)	0.106ꬸ
	Normal	18(58.1)	17(44.7)	35(50.7)	
	Mild to moderate deficiency	2(6.5)	10(26.3)	12(17.4)	
	Toxicity	1(3.2)	0(0)	1(1.4)	
	Total	31(100)	38(100)	69(100)	
>9.2	Severe deficiency	7(20.6)	5(18.5)	12(19.7)	0.016*ꬸ
	Normal	25(73.5)	12(44.4)b	37(60.7)	
	Mild to moderate deficiency	2(5.9)	8(29.6)b	10(16.4)	
	Toxicity	0(0)	2(7.4)	2(3.3)	
	Total	34(100)	27(100)	61(100)	
Alkaline phosphate level					
≤9.1	Severe deficiency	7(21.2)	8(24.2)	15(22.7)	0.172ꬸ
	Normal	24(72.7)	18(54.5)	42(63.6)	
	Mild to moderate deficiency	1(3)	6(18.2)	7(10.6)	
	Toxicity	1(3)	1(3)	2(3)	
	Total	33(100)	33(100)	66(100)	
>9.1	Severe deficiency	10(31.3)	8(25)	18(28.1)	0.020*ꬸ
	Normal	19(59.4)	11(34.4)b	30(46.9)	
	Mild to moderate deficiency	3(9.4)	12(37.5)b	15(23.4)	
	Toxicity	0(0)	1(3.1)	1(1.6)	
	Total	32(100)	32(100)	64(100)	
Smoking Status					
Smoker	Normal	7(77.8)	4(57.1)	11(68.8)	0.758ꬸ
	Mild to moderate deficiency	2(22.2)	2(28.6)	4(25)	
	Toxicity	0(0)	1(14.3)	1(6.3)	
	Total	9(100)	7(100)	16(100)	
Occasional Smoker	Severe deficiency	2(25)	1(25)	3(25)	1.000ꬸ
	Normal	6(75)	3(75)	9(75)	
	Total	8(100)	4(100)	12(100)	
No Smoker	Severe deficiency	15(31.3)	15(27.8)	30(29.4)	0.003*ꬸ
	Normal	30(62.5)	22(40.7)b	52(51)	
	Mild to moderate deficiency	2(4.2)	16(29.6)b	18(17.6)	
	Toxicity	1(2.1)	1(1.9)	2(2)	
	Total	48(100)	54(100)	102(100)	
Comorbidity					
No	Severe deficiency	11(23.4)	7(20.6)	18(22.2)	0.007*ꬸ
	Normal	35(74.5)	18(52.9)b	53(65.4)	
	Mild to moderate deficiency	1(2.1)	8(23.5)b	9(11.1)	
	Toxicity	0(0)	1(2.9)	1(1.2)	
	Total	47(100)	34(100)	81(100)	
Yes	Severe deficiency	6(33.3)	9(29)	15(30.6)	0.629ꬸ
	Normal	8(44.4)	11(35.5)	19(38.8)	
	Mild to moderate deficiency	3(16.7)	10(32.3)	13(26.5)	
	Toxicity	1(5.6)	1(3.2)	2(4.1)	
	Total	18(100)	31(100)	49(100)	
P<0.05, ꬸ Fisher's exact					

On the other hand, in the non-distal radius fracture group, a significantly higher proportion of participants with calcium level >9.2, alkaline phosphate level >9.1, no smokers, and with no comorbidity had mild to moderate Vit D deficiency to the distal radius fracture group. On the contrary, in the distal radius fracture group, a significantly higher proportion of participants who have had similar characteristics mentioned above had normal Vit D levels as compared to the non-distal radius fracture group (p=0.016, 0.020, 0.003 and 0.007, respectively, Table 3).

## Discussion

Vit D deficiency has become a problem affecting people worldwide [1] for clearly identified reasons. Natural food sources need fortification to increase vitamin D content, and hence unfortified food could be a reason for the low Vit D levels worldwide [8]. Similarly, low calcium levels in the body increase the parathyroid hormone (PTH) level, leading to increase catabolism of cholecalciferol [13]. High melanin content in skin, distance from the equator, and seasonal variation also affect the formation of Vit D. All these factors are collectively considered as a cause of low Vit D levels in different parts of the world.

Literature from Pakistan is no different from worldwide, showing a high of 53.5% [4] and 76% [6] in two different studies. This deficiency is reported in the elderly population and includes neonates and pregnant women as well. Our study found that the deficiency and insufficiency pattern was lower than previous studies, 25.4% and 16.9%, respectively, even when comparing rates of the older age group of <41 years the rates were lower (Table 3). A total of 55.4% of our reported patients had normal levels of Vit D, which shows a decrease in the percentage of insufficiency and deficiencies compared to previous data. The cause of this improvement can be attributed to the increased awareness about Vit D deficiency and improvement in food supplements and fortification, which has occurred in recent years.

Hip fractures, distal radius fractures, and spine fractures are commonly reported in the elderly population. However, distal radius fractures are common even in the middle age group and tend to occur early than the other fractures [12]. Most of the reported data is regarding hip fractures, co-related with Vit D deficiency and insufficiency, however, hip fractures are mainly reported in the elderly population. A study by Wang et al. showed 10% of patients presenting with hip fractures showed Vit D deficiency, and 53.33% showed Vit D insufficiency [14]. Wang et al. also stated that those who presented with bilateral hip fractures had very low Vit D levels [14]. Another study from the United States of America showed that 96% of the women with hip fractures had Vit D insufficiency, and 38% had deficiency [15]. Our findings are different from those reported by Oyen et al., which reported Vit D levels of less than 50 nmol/L associated with distal radius fractures [7] although we included the younger population as well, but then again the participants with age greater than 41 years also had lower insufficiency and deficiency rates. Few other studies have shown significantly lower Vit D levels in patients with low-energy distal radius fractures, but the association is insignificantly reported in many other studies [16]. Kim et al. stated that individuals with low Vit D levels improved muscle strength, performance, and fracture healing after Vit D supplementation [17].

Our study showed no significant association between distal radius fracture and vitamin D levels in both age groups of greater and less than 41 years old patients. Mild-moderate deficiency was noted in patients of age ≤41 years, BMI ≤25 kg/m^2^, females with no menopause, albumin >4.2, calcium level >9.2, alkaline phosphate level >9.1, nonsmokers, and with no comorbidity in non-fracture (control) group. At the same time, the distal radius fracture group showed a higher proportion of participants with normal Vit D levels than the non-distal radius fracture group. This gives the impression that Vit D is not significantly associated with distal radius fracture.

To our knowledge, this case-control study is the ﬁrst to compare Vit D levels in distal radius fracture patients and matched controls irrespective of the ages. Male patients have not previously been studied so extensively as most of our patients were male (53.8%). We also looked into other laboratory parameters and serum albumin, calcium levels, and alkaline phosphatase levels, which were not seen in previous studies.

There are certain limitations of the study. The same small size and no knowledge assessment regarding awareness of vitamin D was done in our study as this could have changed the patterns of vitamin D supplementation in recent times. Distal radius fractures usually occur early to other fractures [11] and are considered a precursor of future hip and vertebral bone fractures especially in the elderly population [18, 19]. Haentjens et al. [20] also reported that the relative risk of hip fractures after distal radius fracture in the elderly population was more significant in men than in women. Therefore, an effort must be made to identify risk factors to reduce future hip and vertebral bone fractures risks as early in life as possible.

We, therefore, recommend that, since previously an association was found between Vit D and other fractures, a study on a larger scale should be conducted to re-assess the current situation and a longer follow-up of patients, which can also highlight the importance of Vid D in the healing of fractures and prevention of other fractures.

## Conclusions

In conclusion, our study indicates that Vitamin D inadequacy is not associated with distal radius fractures in women and men. Differences in Vitamin D levels between patients and controls were independent of differences in other variables such as age, serum calcium, alkaline phosphatase, albumin levels, and smoking history. At the same time, the association was significant with low BMI and high sunlight exposure time.
